# Supplementary material for: Causal Relationship between Aging and Anorexia Nervosa: A White-Matter-Microstructure-Mediated Mendelian Randomization Analysis
Source: Biomedicines. 2024 Aug 16;12(8):1874. doi: 10.3390/biomedicines12081874 (PMC11351342; doi:10.3390/biomedicines12081874)
Supplement: Supplementary file 1 [file biomedicines-12-01874-s001.zip › Supplementary Figure/Figure S8/Leaveoneout plot of MR analysis for TL to ubm-b-1465.pdf]

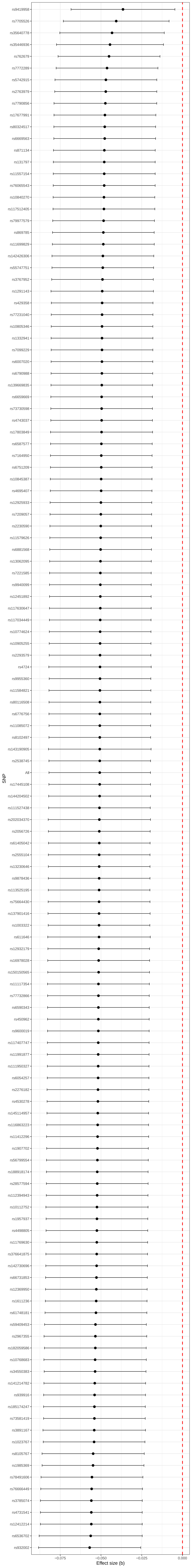
rs9419958
rs7705526
rs35640778
rs35446936
rs762679
rs7772289
rs5742915
rs2763979
rs7790856
rs17677991
rs80324517
rs6669563
rs871134
rs131797
rs11557154
rs76065543
rs10840270
rs117512405
rs79977579
rs869785
rs11699829
rs142426306
rs55747751
rs3767952
rs1291143
rs429358
rs77231040
rs10805346
rs1332941
rs7099229
rs6007020
rs6790988
rs139669835
rs6659669
rs73730598
rs4743037
rs17803849
rs6587577
rs7164950
rs6751209
rs10845387
rs4695407
rs12925933
rs7209057
rs2230590
rs11579626
rs6881568
rs13062095
rs7221585
rs9940099
rs12451892
rs117630647
rs117034449
rs10774624
rs10905255
rs2293579
rs4724
rs9955360
rs11584821
rs80116508
rs6776756
rs11085072
rs8102497
rs143190905
rs2538745
A8
rs17445108
rs144204502
rs111527438
rs202034370
rs2056726
rs61405042
rs2555104
rs13230646
rs9878436
rs113525195
rs75664430
rs137901416
rs1003322
rs611646
rs12932179
rs16978028
rs150150565
rs11117354
rs77732866
rs6590343
rs450962
rs9600019
rs117407747
rs11991877
rs111950327
rs6054257
rs2276182
rs4530278
rs145114957
rs116863223
rs11412296
rs1907702
rs56799554
rs188918174
rs28577594
rs112394943
rs10112752
rs1957937
rs4498805
rs11769630
rs376641875
rs142730696
rs66731853
rs12369950
rs1611236
rs61748181
rs59409453
rs2967355
rs182059586
rs10768683
rs34550363
rs141214782
rs939916
rs185174247
rs73581419
rs3891167
rs1023767
rs8105767
rs1985369
rs78491606
rs76666449
rs3785074
rs4731541
rs12412214
rs6536702
rs932002
SNP
-0.075
-0.050
-0.025
0.000
Effect size (b)
